# The mutation spectrum in Indian patients with Gaucher disease

**DOI:** 10.1186/gb-2011-12-s1-p26

**Published:** 2011-09-19

**Authors:** Vartika Bisariya, Pramod K Mistry, Jun Liu, Madhumita Roy Chaudhari, Neerja Gupta, Madhulika Kabra

**Affiliations:** 1 Genetics Division, Department of Pediatrics, All India Institute of Medical Sciences, New Delhi, India; 2 Yale University School of Medicine, New Haven, USA

## 

Gaucher disease is the most common lysosomal storage disorder. It results from an inherited deficiency of the enzyme glucocerebrosidase (GBA); accumulation of the substrate of this enzyme has many clinical manifestations. Since the discovery of the *GBA* gene, more than 200 mutations have been identified, but only a handful of mutations are recurrent (L444P, N370S, IVS2, D409H and 55Del). To determine the spectrum of mutations in the Indian population, we performed mutational screening in children with Gaucher disease.

Twenty-four patients from twenty families were enrolled in this study, after written informed consent was obtained. The diagnosis of Gaucher disease was based on mandatory clinical and biochemical analysis. An initial screening for five common mutations was carried out using PCR-RFLP. Patients who were negative for common mutations were screened by sequencing exons 9 to 11 (a mutation hotspot region) [1].

We identified common mutations (L444P, N370S, IVS2 and D409H [2], and 55Del [3]) in approximately 50% of the patients. L444P (c.1448T>C) was the most frequently identified, followed by D409H in our patients. Western data shows that N370S is the most common mutation in Romanian patients [4]. One polymorphism (E340K) was identified in two patients who were compound heterozygotes for A456P/R463C and S237F/A269P, respectively. Our data highlight the spectrum of mutations that lead to Gaucher disease in the Indian population.
